# miR-29a-5p Alleviates Traumatic Brain Injury- (TBI-) Induced Permeability Disruption via Regulating NLRP3 Pathway

**DOI:** 10.1155/2021/9556513

**Published:** 2021-11-28

**Authors:** Aijun Zhang, Youming Lu, Lei Yuan, Pengqi Zhang, Dongdong Zou, Fance Wei, Xin Chen

**Affiliations:** Department of Neurosurgery, The Affiliated Sixth People's Hospital, Shanghai Jiaotong University, Shanghai 200233, China

## Abstract

**Objective:**

Inactivation of NLRP3 inflammasome plays a role in reducing the permeability of endothelial cells and improving blood-brain barrier (BBB) dysfunction following traumatic brain injury (TBI). However, the mechanism controlling NLRP3 inflammasome activation remains unclear. This study is aimed at defining the role of miR-29a-5p in NLRP3 inflammasome activation and permeability of endothelial cells under TBI.

**Methods:**

The scratch injury model on brain bEnd.3 microvascular endothelial cells was used as in vitro TBI model cells. Effects of miR-29a mimics and inhibitors on TBI model cells were observed by examining their action on FITC, TEER, and protein contents of ZO-1 and occludin, and cell permeability-associated protein. Luciferase reporter assay evaluated miR-29a-5p targeting to NLRP3. ELISA examined of IL-1*β* and IL-18 levels. miR-29a-5p mimic was injected into TBI mouse and its effect on BBB, indicated by Evans blue (EB) staining assay and cerebral water content, and NLRP3 activation was examined.

**Results:**

miR-29a-3p and miR-29a-5p mimics decrease the concentration of FITC, and increase TEER and the protein contents of ZO-1 and occludin in TBI model cells. miR-29a-5p silencing disrupted the permeability of mouse bEnd.3 cells. miR-29a-5p targets to NLRP3 through the binding on its 3′UTR and negatively regulates its expression in TBI model cells. NLRP3 inhibition and miR-29a-5p silencing together caused significantly decreased FITC concentration and increased TEER value and release of IL-1*β* and IL-18. miR-29a-5p mimic alleviated the BBB and cerebral water content and inactivates NLRP3 in the mouse TBI model.

**Conclusions:**

miR-29a-5p mimics protect TBI-induced increased endothelial cell permeability and BBB dysfunction via suppressing NLRP3 expression and activation.

## 1. Introduction

Traumatic brain injury (TBI) is a subset of acquired brain damage and causes a high mortality and disability rate for patients, and, in the surviving patients, impairments in neurological function [1]. Understanding the mechanisms of pathology and finding reliable therapeutic targets is essential for both physicians and patients. The encouraging discovery is that posttraumatic permeability disruption of the blood-brain barrier (BBB) is responsible for neuron loss and brain function alteration in TBI [2–4]. In this case, the semipermeable BBB, constituted by cerebral endothelial cells, can no longer carry out its protective function to keep the harmful content away from the central nervous system [5]. This is largely due to a significant downregulation in the expression of tight junctional proteins, principally ZO-1 and occludin, on endothelial cells [6–8]. BBB permeability, therefore, is recognized as one of the significant factors for the progression of brain damage and the response to therapy [1]. Consequently, research centered on the underlying mechanism controlling endothelial cell function and BBB permeability is in progress.

Pieces of evidence have recently identified inflammasome formation, and activation is responsible for BBB dysfunction following TBI [9–11]. NLRP3 (nucleotide-binding domain leucine-rich repeats family protein 3)/Caspase-1 is such an inflammasome pathway related to posttraumatic permeability disruption of the BBB via mediating neuroinflammatory response [12]. Such NLRP3/Caspase-1 inflammasome activation following TBI subsequently induced the generation of the proinflammatory cytokines IL-1*β* and IL-18 and resulted in neuron loss and neurological disorder [13, 14]. Herein, efforts on gene expression inhibition and inactivation of NLRP3 are increasingly made to be a promising therapeutic strategy for TBI by researchers [10, 15, 16].

MicroRNAs (miRNAs) are increasingly seen as key controllers for gene expression in the mammal using a way of posttranscriptional regulation through paired binding to target mRNA 3′UTR (untranslated regions) and thereby inhibit gene-associated signaling pathway activation [17]. miRNAs aberrantly expressed following TBI and were involved in regulating neuron death, BBB dysfunction, and inflammatory response [18–20]. miR-29a is a multifunctional miRNA in regulating the pathological processes of many diseases via controlling cell death, differentiation, and inflammatory reaction [21–23]. In the brain, miR-29a appears to be a protector with multifunctions [24–26]. However, the role of miR-29a in posttraumatic endothelial cell function and BBB permeability is rarely known. Therefore, our experiments in this project were aimed at exploring the action of miR-29a-5p and miR-29a-3p on the permeability of TBI model cells.

## 2. Materials and Methods

### 2.1. Cell Culture

Mouse bEnd.3 cells was provided by Shanghai Biology Institute (Shanghai, China) and cultured in DMEM with the extra addition of 10% FBS, 2 mM l-glutamine, and 1% penicillin/streptomycin (Solarbio, China). A continuous culture condition of 5% CO_2_ atmosphere and 37°C was supplied for cell experiments.

### 2.2. In Vitro Model

TBI model cells were established by scratch bEnd.3 cells method. bEnd.3 cells were grown in a 24-well plate until the formation of the cell monolayer. Scratch injury of a 0.5 mm wide gap was made with a sterile 26G syringe needle. The scraped cells were rinsed off with PBS. bEnd.3 cells in the control group were with no scratch injury.

### 2.3. ELISA

The medium or serum content of IL-18 and IL-1*β* was analyzed using the ELISA method with ELISA kits basing on the manufacturer's instruction.

### 2.4. QRT-PCR

Total RNA was made by using TRIzol reagent (Invitrogen, USA) supplementary with RNase inhibitor. For the first step of the qRT-PCR procedure, a total RNA sample was added to the reverse transcriptional reaction solution of the cDNA synthesis kit to produce cDNA. Then, the cDNA product was added to a real-time PCR reaction solution (SYBR™ Green Master Mix Applied Biosystems™) and amplified based on the primers with the reaction conditions of 95°C for 10 minutes, 40 cycles of 95°C for 15 seconds, and 60°C for 45 seconds. Primer sequences were listed in Supplemental file 1. Gene expressions were calculated by the 2^−*ΔΔ*Ct^ method and employed U6 or GAPDH as the internal control.

### 2.5. Western Blot

Total protein was made by using RIPA lysis buffer added with protease inhibitor cocktail (Roche, Germany). After a quantitative analysis using the BCA protein assay kit, test protein samples with equal amounts were subjected to 10-12% SDS-PAGE. A transfer operation then was performed to transfers the protein from the gel to a nitrocellulose membrane (Millipore, USA) and then was going through 1 hr of blockage with 5% nonfat dry milk, 12 hr of incubation with primary antibodies (Seen in Supplemental file 2), and then 1 hr of incubation with secondary antibody (Beyotime, China). Protein blotting was detected using an enhanced chemiluminescence system (Beyotime, China). The protein expression was relative to GAPDH. The primary antibodies were list in.

### 2.6. Cell Permeability Detection

Cells were inoculated onto the upper chamber of the plate and grown to the formation of cell monolayer, during which the medium should be changed every day. FITC-Dextran was mixed into the medium at the last change of medium and maintained for 24 hr. Plates were placed in a microplate reader (Pulangxin, China) to analyze the intensity of FITC fluorescence at 490 nm.

### 2.7. TEER Assay

TEER value of bEnd.3 cell monolayer was analyzed using Millcell ERS-2 Voltohmmeter (Millipore, USA). Cells were and grown to monolayer at a 24-well plate and subjected to TEER value detection as the protocols suggested by the manufacturer. Calculation formula was TEER value (*Ω*·cm2) = TEER (*Ω*) × surface area (0.6 cm2).

### 2.8. Luciferase Reporter Assay

An amplified procedure was performed on the sequence of NLRP3 3′UTR containing the binding site of miR-29a-5p followed by a clone operation into luciferase reporter pGL3 vector (WT 3′UTR). The clone of sequence with the mutant binding sites was as control (Mutant 3′UTR). The recombinant luciferase reporter vectors were cotransfected with the miR-29a-5p inhibitor or mimic into bEnd.3 cells. Luciferase activity of each cell treatment was examined after 48 hr of transduction with the Dual-GLO Luciferase Assay Kit (Promega, USA) on a plate reader.

### 2.9. TBI Mouse Model

Eighteen C57B6/J mice (male, aged 8-10 weeks) were randomly divided into three groups basing on different treatments: control mice were received sham operations, negative control for mimic (miNC) TBI mice were injected with miNC, and mimic TBI mice were injected with miR-29a-5p mimic. After TBI, mice were raised for another 48 hr before sacrifice for tissue examination. The sham mice received the same operation except for impact. All performances on animal experiments complied with the Guide for the Care and Use of Laboratory Animals and approved by the Ethics Committee of Shanghai Sixth People's Hospital East Affiliated to Shanghai University of Medicine & Health Sciences.

### 2.10. Mouse Brain Water Content

The difference in weight between the wet brain and dry brain was used to evaluate the brain water content of the treated mice in each group. WW represents the wet weight and was weighted when fresh brains obtainment. DW represents the dry weight and was weighted after 72 hr of drying at 70°C. The calculation formula is Brain water content = [WW − DW]/WW × 100%.

### 2.11. Evans Blue Evaluates BBB Permeability

After intraperitoneal injection with sodium pentobarbital (60 mg/kg), mice were received right femoral vein infusion with 2% evans blue dye (5 ml/kg in saline) using a PE-50 catheter for 30 min. Another 15 min of heart perfusion with saline was worked to remove intravascular EB dye. Brain tissues were removed for the detection of EB dye permeating from BBB at a fluorescent plate reader. The intensity of fluorescence was measured at 630 nm. EV value was calculated as *μ*g per 1 g of the brain.

### 2.12. H & E Staining Assay

Histological changes in the brain of TBI mice were visualized by HE staining. In brief, the brain was removed and immediately immersed in 4% paraformaldehyde. Then, paraffin sections were made from these fixed tissues using a microtome and went through deparaffin and rehydration before HE staining.

### 2.13. Statistical Analysis

Data statistic was conducted on a version 7.0 software of GraphPad Prism (USA). Difference analysis between groups or among multiple groups used the *t*-test and one-way analysis of variance. Data were expressed as mean ± SD of at least three samples and triplicates were made if necessary. The *p* value of less than 0.05 was considerable statistical significance on the difference.

## 3. Results

### 3.1. miR-29a-3p or miR-29a-5p Mimics Alleviated TBI-Induced Permeability Disruption in Mouse bEnd.3 Cells

**Figure 1:**
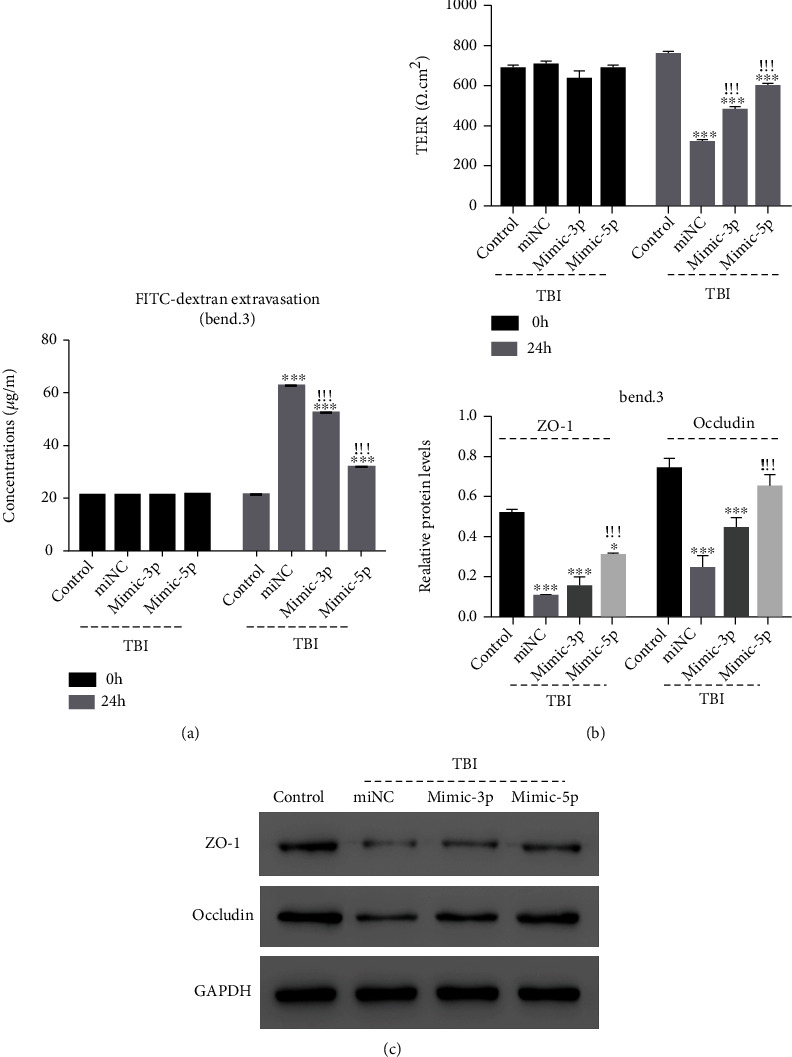
miR-29a-3p and miR-29a-5p mimics mitigated permeability disruption in TBI model cells. (a) The concentration of FITC, (b) the TEER value, and (c) ZO-1 and occludin expression. ^∗^*p* < 0.05, ^∗∗∗^*p* < 0.001 compared with the control; ^!!!^*p* < 0.001 compared with miNC.

To determine the role of miR-29a-3p and miR-29a-5p in the permeability disruption of TBI model cells, they were overexpressed in the mouse microvascular endothelial bEnd.3 cells before the establishment of the TBI model (Figure S1). As shown in Figure 1(a), both miR-29a-3p and miR-29a-5p overexpression significantly decreased the FITC concentration in TBI model cells compared with miNC at 24 h postmimics transfection. The TEER and the protein contents of ZO-1 and occludin of TBI model cells were recovered by both miR-29-3p and miR-29a-5p overexpression, especially for miR-29a-5p (Figures 1(b) and 1(c)). Then, miR-29a-5p was chosen for the follow-up subjects.

### 3.2. miR-29a-5p Silencing Disrupted the Permeability of Mouse bEnd.3 Cells

**Figure 2:**
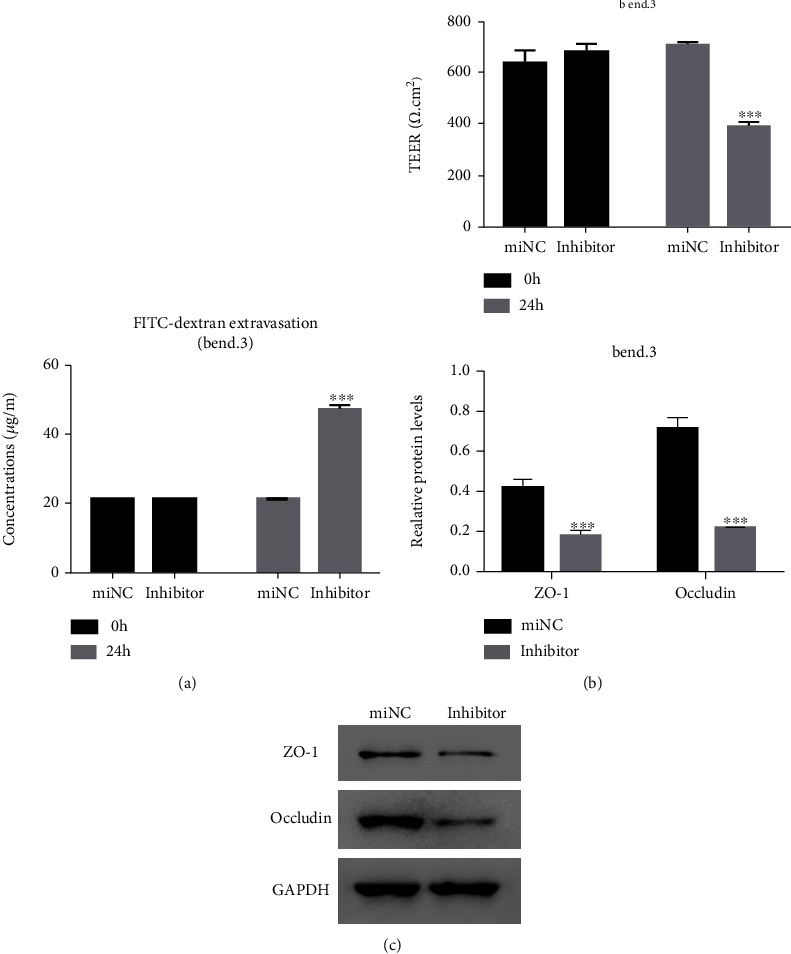
miR-29a-5p silencing disrupted the permeability of mouse bEnd.3 cells. (a). FITC concentration was increased by miR-29a-5p inhibitor. (b) The TEER value was reduced by miR-29a-5p inhibitor. (c) ZO-1 and occludin were downregulated by the miR-29a-5p inhibitor. ^∗∗∗^*p* < 0.001 compared with miNC.

To define the role of miR-29a-5p in the permeability of cerebral epithelial cells, miR-29a-5p was silenced in bEnd.3 cells. Results showed that FITC concentration was increased (Figure 2(a)), TEER value was decreased, and ZO-1 and occludin were expressed at lower levels (Figures 2(b) and 2(c)).

### 3.3. miR-29a-5p Inhibited the Expression of NLRP3 through Binding on Its 3′UTR

**Figure 3:**
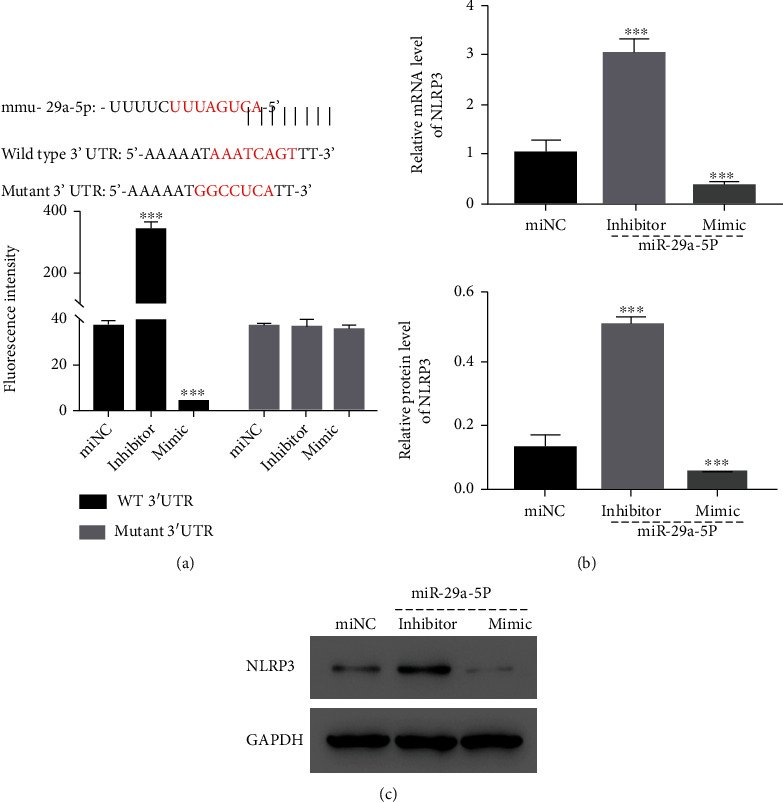
NLRP3 expression was suppressed by miR-29a-5p via the direct binding to its 3′UTR. (a) The paired sequences between miR-29a-5p and NLRP3 3′UTR were marked as red (upper). (b, c) Relative mRNA and protein levels of NLRP3 were determined using qRT-PCR and western blot, respectively, in the indicated groups of bEnd.3 cells. ^∗∗∗^*p* < 0.001 compared with miNC.

By the prediction in bioinformatics software, miR-29a-5p had a potential binding site to NLRP3 3′UTR (Figure 3(a)-upper part). The luciferase reporter analysis showed that the luciferase activity of reporter constructed with the binding site could be enhanced by miR-29a-5p silencing and suppressed by miR-29a-5p overexpression, a reporter with the mutant binding site was unaffected, suggesting the possible regulating role of miR-29a-5p on NLRP3 expression (Figure 3(a)-lower part). Therefore, such a possibility was further demonstrated by the results of qRT-PCR and western blot where NLRP3 mRNA and protein levels were upregulated in bEnd.3 cells with miR-29a-5p silencing but lacked in cells with miR-29a-5p overexpression (Figures 3(b) and 3(c)).

### 3.4. NLRP3 Content of TBI Model Cells Was Suppressed by miR-29a-5p Mimic

**Figure 4:**
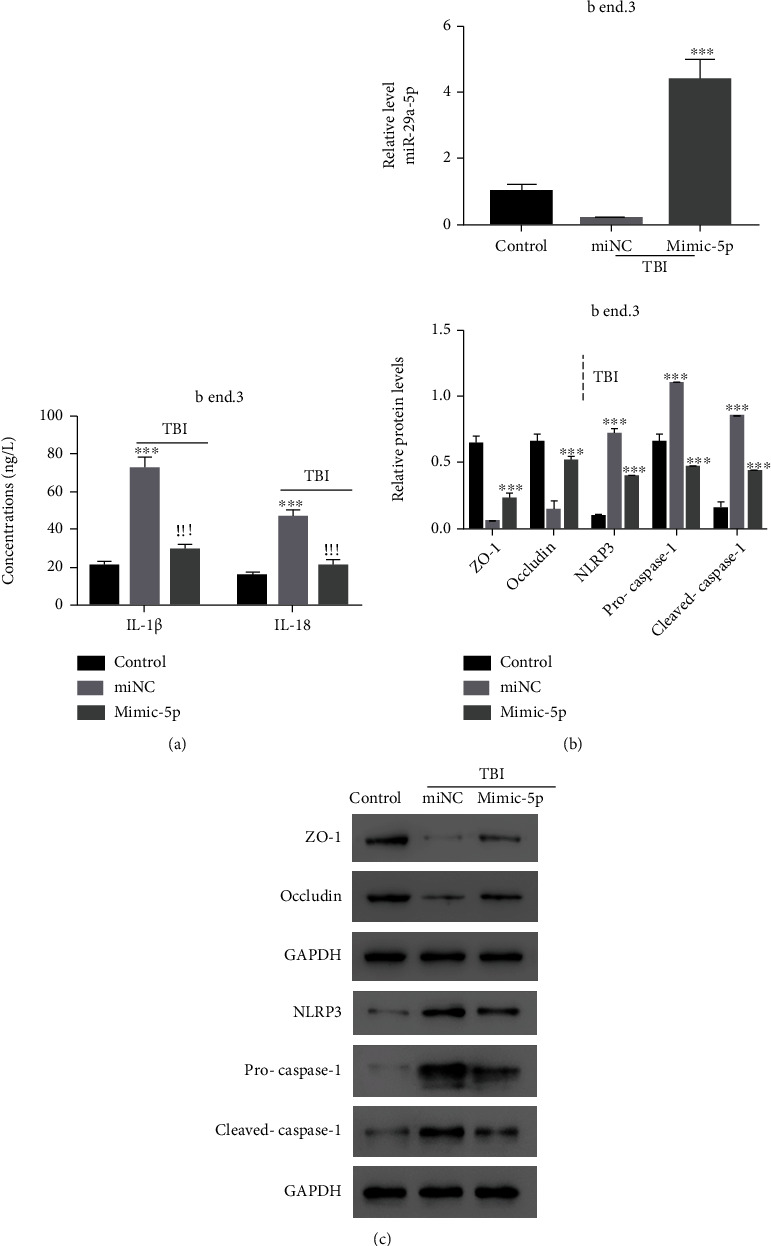
miR-29a-5p overexpression inhibited NLRP3 expression in TBI model cells. (a). miR-29a-5p overexpression reduced IL-1*β* and IL-18 concentration in TBI model cells. (b) miR-29a-5p enriched in TBI model cells transduced with mimic-5p. (c) The protein levels of ZO-1, occludin, NLRP3, Pro-Caspase-1, and Cleaved Caspase-1 were determined using western blot. ^∗∗∗^*p* < 0.001 compared with the control, ^!!!^*p* < 0.001 compared with miNC.

miR-29a-5p mimic transfection drove the suppression of IL-1 *β* and IL-18 release from TBI model cells (Figure 4(a)) and greatly upregulated miR-29a-5p expression level in the TBI model cells (Figure 4(b)). In such TBI model cells with miR-29a-5p overexpressed, ZO-1 and occludin expressed at a high level, and NLRP3/Caspase-1 inflammasome activation-related gene expression including NLRP3, Pro-Caspase 1, and Cleaved Caspase-1 was significantly downregulated compared with cells treated with miNC (Figure 4(c)).

### 3.5. The NLRP3 Inhibitor CY-09 Rescued the Role of miR-29a-5p

**Figure 5:**
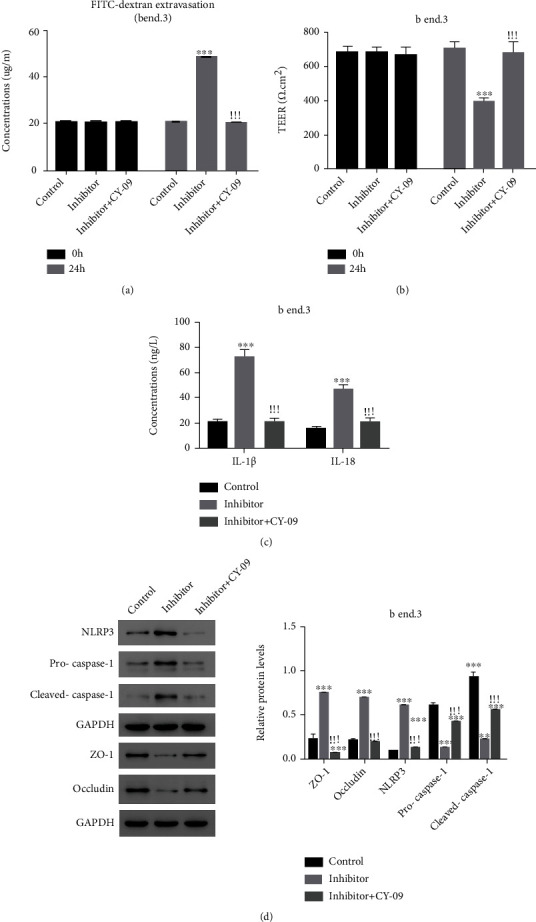
The NLRP3 inhibitor CY-09 rescued the role of miR-29a-5p in mouse bEnd.3 cells.. (a) The concentration of FITC, (b) the TEER value, (c) the concentration of IL-1 *β* and IL-18, and (d) the protein contents of NLRP3, Pro-Caspase-1, Cleaved Caspase-1, ZO-1, and occludin were examined in indicated groups of cells. ^∗∗∗^*p* < 0.001 compared with the control, ^!!!^*p* < 0.001 compared with the inhibitor.

To determine the involved NLRP3 in the role of miR-29a-5p, CY-09, an antagonist for NLRP3, and miR-29a-5p inhibitor together treated mouse bEnd.3 cells and their effect on cell permeability was observed at 24 h posttreatment. As shown in Figures 5(a)–5(c), compared with miR-29a-5p silencing, NLRP3 inhibition and miR-29a-5p silencing together caused the decreased FITC concentration, the increased TEER value, and the decreased IL-1*β* and IL-18 concentration. Furthermore, bEnd.3 cells with the coprocessing of NLRP3 inhibitor and miR-29a-5p inhibitor expressed lower levels of NLRP3, Pro-Caspase-1, and Cleaved Caspase-1 and were with higher levels of ZO-1 and occludin compared with cells with miR-29a-5p alone (Figure 5(d)). The above results indicate that miR-29a-5p may control the BBB via regulating NLRP3.

### 3.6. miR-29a-5p Mimic Alleviated the TBI-Induced Permeability Disruption In Vivo

**Figure 6:**
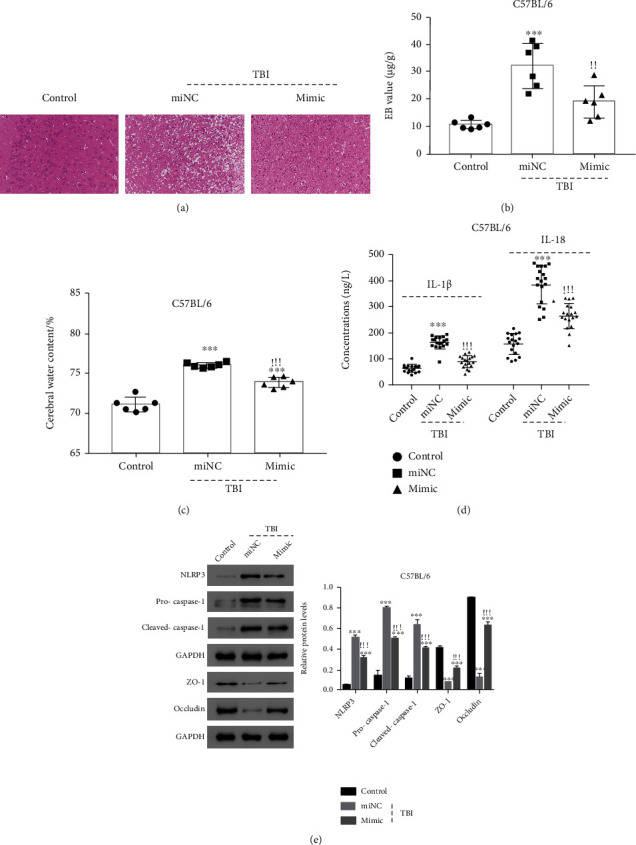
miR-29a-5p mimic injection induced the alleviation of BBB permeability in the TBI mouse model. (a) Brain morphology was evaluated using HE staining (*n* = 6). Amplification of 200x. (b) The permeability of mouse BBB was analyzed using Evans blue (EB) staining assay (*n* = 6). (c) Cerebral water content was examined (*n* = 6). (d) Serum levels of IL-1 *β* and IL-18 were analyzed using ELISA (*n* = 6). (e) The protein levels of NLRP3, Pro-Caspase-1, Cleaved Caspase-1, ZO-1, and occludin were determined using western blot (*n* = 6). ^∗∗∗^*p* < 0.001 compared with the control, ^!!!^*p* < 0.001 compared with miNC.

By results of HE staining, miR-29a-5p mimic injection alleviated the pathomorphological changes of TBI mouse (Figure 6(a)). Except that, miR-29a-5p mimic injection reduced the permeability of BBB (Figure 6(b)) and the cerebral water content in TBI mouse (Figure 6(c)). The serum levels of IL-1*β* and IL-18 were also reduced after miR-29a-5p mimic injection (Figure 6(d)); moreover, the similar results were also obtained by using mouse brain tissues (Figure S2). Western blot results showed that the brain tissue of TBI mouse with miR-29a-5p mimic injection expressed lower levels of NLRP3, Pro-Caspase-1, and Cleaved Caspase-1 and was with higher levels of ZO-1 and occludin compared with TBI mice with miNC injection (Figure 6(e)).

## 4. Discussion

Recently, aberrant expression levels of miRNA are recognized as an important regulator in TBI [27]. Our research concentrated on miR-29a and explored its role in endothelial cell permeability. Shi et al. thought of miR-29a as the prognosis marker for gliomas [28]. Ouyang et al. revealed the positive role of astrocyte-enriched miR-29a in forebrain ischemia [26]. Beyond that, miR-29a is also known as a protector in maintaining functions of endothelial cells [29, 30]. At the same time, peripheral blood miR-29a could be upregulated in patients with severe TBI at a particular time point after injury [31]. This dynamic change in expression further arouses our research interest to investigate the involved miR-29a in the permeability of cerebral endothelial cells under TBI.

Normally, BBB is mainly maintained by a certain level of the tight junctional proteins and benefits for the brain by enabling nutrients supplication and avoiding damage from harmful substances [1]. The induced-high expression of tight junctional protein ZO-1 and occludin by miRNAs closely correlated with the maintenance of BBB permeability [32, 33]. Our data also approve this regulation mechanism, which showed that the protein contents of ZO-1 and occludin were increased by miR-29a overexpression in TBI model cells along with the reduced cell permeability. Herein, we identify the key function of miR-29a in alleviating permeability disruption in TBI model cells.

Studies flourishing explored the treatment for TBI based on the inhibition of NLRP3 inflammasomes activation, and this strategy was regarded as a potentially promising treatment strategy for TBI [13, 16]. The key role of miRNA in controlling NLRP3 expression may provide an alternative way to suppress NLRP3 inflammasome activation, which has been demonstrated in animal and cell experiments [34–36]. Our data specifically stress that NLRP3 is a targeted molecule of miR-29a-5p and its expression can be negatively regulated by miR-29a-5p in TBI model cells. So, this is also a piece of strong evidence to demonstrate the effectiveness of miR-29a overexpression in treating TBI. Our result of in vivo experiments with the injection of miR-29a-5p mimic into TBI mouse is a good illustration of this.

## Data Availability

The datasets used and/or analyzed during the current study are available from the corresponding author on reasonable request.

## Abbreviations

TBI: Traumatic brain injury
BBB: Blood-brain barrier
miRNA: MicroRNA
UTR: Untranslated regions.

## Supplementary Materials

Supplementary Materials Figure S1: miR-29a-3p or miR-29a-5p mimics significantly improved the expression of miR-29a-3p or miR-29a-5p, respectively, in mouse bEnd.3 cells. ^∗∗∗^*p* < 0.001 compared with miNC. Figure S2: the miR-29a-5p mimic suppressed the protein expression of IL-1*β* and IL-18 in mouse brain tissues of TBI. ^∗∗^*p* < 0.01 vs. the control, ^∗∗∗^*p* < 0.001 vs. the control; ^!!^*p* < 0.01 vs. miNC, ^!!!^*p* < 0.001 vs. miNC.
