# Supplementary material for: miR-29a-5p Alleviates Traumatic Brain Injury- (TBI-) Induced Permeability Disruption via Regulating NLRP3 Pathway
Source: Dis Markers. 2021 Nov 28;2021:9556513. doi: 10.1155/2021/9556513 (PMC8645411; doi:10.1155/2021/9556513)
Supplement: Supplementary Materials — Figure S1: miR-29a-3p or miR-29a-5p mimics significantly improved the expression of miR-29a-3p or miR-29a-5p, respectively, in mouse bEnd.3 cells. ∗∗∗p < 0.001 compared with miNC. Figure S2: the miR-29a-5p mimic suppressed the protein expression of IL-1β and IL-18 in mouse brain tissues of TBI. ∗∗p < 0.01 vs. the control, ∗∗∗p < 0.001 vs. the control; !!p < 0.01 vs. miNC, !!!p < 0.001 vs. miNC. [file 9556513.f1.pdf]

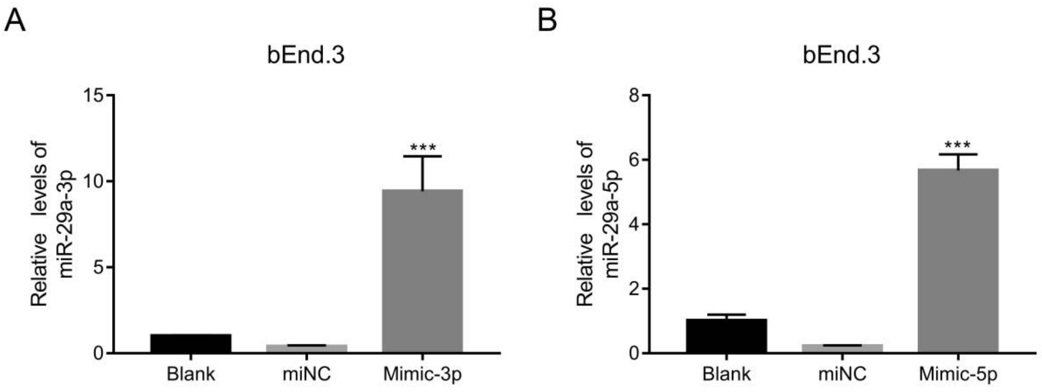

Figure S1: miR-29a-3p or miR-29a-5p mimics significantly improved the expression of miR-29a-3p or miR-29a-5p respectively in mouse bEnd.3 cells. *** $p < 0.001$ compared with miNC.

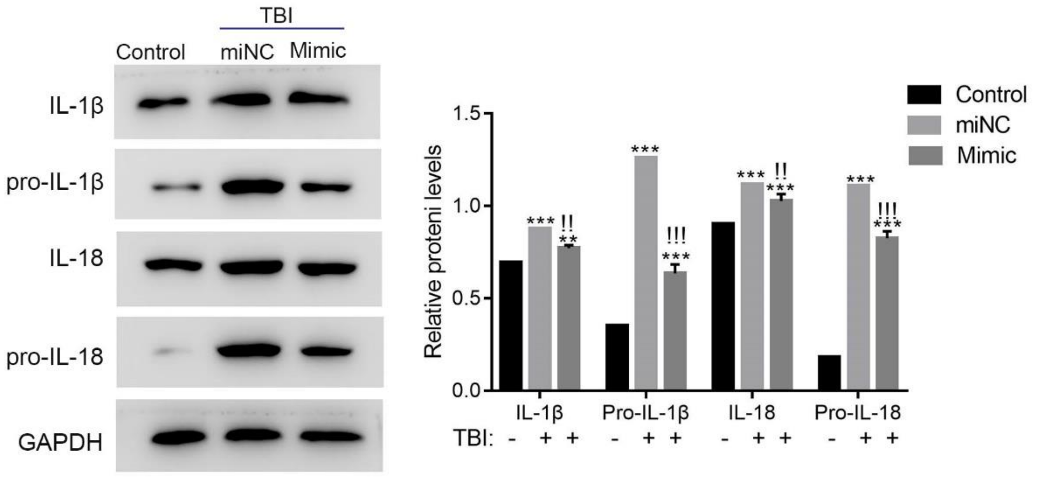

Figure S2: The miR-29a-5p mimic suppressed the protein expression of IL-1β and IL-18 in mouse brain tissues of TBI. ** $p<0.01$ vs control, *** $p<0.001$ vs control; !! $p<0.01$ vs miNC, !!! $p<0.001$ vs miNC.
